# The Sugar-Signaling Hub: Overview of Regulators and Interaction with the Hormonal and Metabolic Network

**DOI:** 10.3390/ijms19092506

**Published:** 2018-08-24

**Authors:** Soulaiman Sakr, Ming Wang, Fabienne Dédaldéchamp, Maria-Dolores Perez-Garcia, Laurent Ogé, Latifa Hamama, Rossitza Atanassova

**Affiliations:** 1 Institut de Recherche en Horticulture et Semences, Agrocampus-Ouest, INRA, Université d′Angers, SFR 4207 QUASAV, F-49045 Angers, France; ming.wang@agrocampus-ouest.fr (M.W.); maria-dolores.perez-garcia@agrocampus-ouest.fr (M.-D.P.-G.); laurent.oge@agrocampus-ouest.fr (L.O.); latifa.hamama@agrocampus-ouest.fr (L.H.); 2 Equipe “Sucres & Echanges Végétaux-Environnement”, Ecologie et Biologie des Interactions, Université de Poitiers, UMR CNRS 7267 EBI, Bâtiment B31, 3 rue Jacques Fort, TSA 51106, 86073 Poitiers CEDEX 9, France; Fabienne.Dedaldechamp@univ-poitiers.fr (F.D.); Rossitza.Atanassova@univ-poitiers.fr (R.A.)

**Keywords:** sugar, pathway, sensing, crosstalk, regulation, hormone, nitrogen

## Abstract

Plant growth and development has to be continuously adjusted to the available resources. Their optimization requires the integration of signals conveying the plant metabolic status, its hormonal balance, and its developmental stage. Many investigations have recently been conducted to provide insights into sugar signaling and its interplay with hormones and nitrogen in the fine-tuning of plant growth, development, and survival. The present review emphasizes the diversity of sugar signaling integrators, the main molecular and biochemical mechanisms related to the sugar-signaling dependent regulations, and to the regulatory hubs acting in the interplay of the sugar-hormone and sugar-nitrogen networks. It also contributes to compiling evidence likely to fill a few knowledge gaps, and raises new questions for the future.

## 1. Introduction 

Plant growth and development are regulated by many factors including light, temperature, water, sugars, and plant hormones. As plants are autotrophic organisms, they produce their carbon skeletons through photosynthesis in the form of sugars that serve as structural components and energy sources throughout their life [1]. Sugars are also signaling entities; therefore, perception and management of sugar levels by plants are critical for their survival. Plants have evolved a complex mechanistic system to sense different sugars, including sucrose, hexoses, and trehalose. These sugars elicit adequate responses, some of which are specific to the type of sugar [2,3,4,5,6]. Sugar signaling is also a mechanism that plants use to integrate various internal and external cues to achieve nutrient homeostasis, mediate developmental programs, and orchestrate stress responses [6]. In addition, the carbon and nitrogen metabolisms are closely connected with each other at almost all stages of plant growth and development. The coordination and the integration of the C and N metabolic and signaling pathways appear crucial for the improvement of plant performances [7,8]. Besides nutrients, plant hormones play a determining role in plant development and are key transmitters of developmental programs [9]. In addition, the interaction between sugars and plant hormones is orchestrated to finely regulate the main biological processes throughout the plant life cycle. In this context, and with a view to understanding the mechanisms involved in the sugar and plant hormone networks, it is important to decipher their crosstalk with sugars during plant development. The present review emphasizes the emerging understanding of the main sugar signaling pathways in plants and the array of molecular and biochemical mechanisms that mediate the effects of the sugar signal. To provide a comprehensive idea about the relevance of the sugar-signaling-dependent regulation of plant functioning, this review also addresses the interplay between sugar, nitrogen, and the main plant hormones, while focusing on certain biological contexts. This work also underlines a few emerging linker molecular actors that deserve to be further investigated.

## 2. Sugar Signaling Pathways

### 2.1. Disaccharide Signaling Pathways

**Figure 1:**
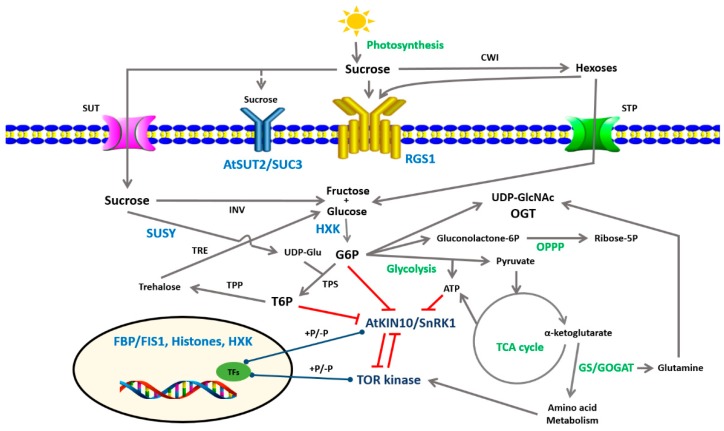
Schematic representation of the sugar metabolism (green) and signaling (blue) pathways. Sugar sensing involves for example glucose sensors (HXK: Hexokinase, OGT: O-Glucose *N*-acetyl transferase); fructose sensors (i.e., FBP/FIS1, fructose-1,6-bisphosphatase), sucrose and hexose sensors (RGS1, regulator of G-protein signaling), and putative sucrose sensors (SUSY, SUcrose SYnthase, putative sucrose transporter AtSUT2/SUC3). Downstream of sugar perception are two energy sensors: AtKIN10/SnRK1 (sucrose-non-fermentation-related protein kinase1) and TOR-kinase (target of rapamycin kinase). CWI: cell wall invertase; G6P: glucose 6-phosphate; GS/GOGAT: glutamine synthetase/glutamate oxoglutarate aminotransferase; INV, invertase; OPPP: oxidative pentose phosphate pathway; STP: Sugar transport protein; SUT: sucrose transporter; TCA cycle: Tricarboxylic acid cycle; TFs: transcription factors; TPS: T6P synthase; TPP: trehalose 6P phosphatase; T6P, trehalose 6-phosphate; UDPGlc: Uridine diphosphate glucose; UDPGlc-NAC: UDP *N*-acetylglucosamine.

Sucrose is the main sugar for systemic source-to-sink transport in plants. The dual role of sucrose, i.e., as an energy source and a signaling entity, was evidenced by many experiments showing that non-metabolizable sucrose analogs could mimic the effect of sucrose while its derivative hexoses (glucose and fructose) have very low efficiency [10,11,12,13]. The best-known example is the sucrose-specific down-regulation of *BvSUT1* (*Beta vulgaris* Sucrose Transporter 1), encoding the phloem-located proton-sucrose symporter, which is neither elicited by hexoses nor affected by mannoheptulose, a hexokinase inhibitor [10]. By doing so, sucrose signaling regulates the expression of its own transporter at the site of phloem loading and thereby may control photo-assimilated partitioning between source and sink. Sucrose signaling controls not only plant metabolism but also plant development [6,14,15,16,17,18]. However, the mechanism of sucrose perception remains unknown. Barker et al. (2000) proposed the plasma membrane sucrose transporter AtSUT2/SUC3 as a putative sucrose sensor (Figure 1) [19]. AtSUT2/SUC3 shares structural features with the yeast glucose sensors Snf3 and Rgt2. The tonoplast low-affinity sucrose transporter SUT4 that interacts with five cytochrome b5 family members may also mediate sugar signaling [20]. Downstream of sucrose sensing, calcium, calcium dependent-protein kinases (CDPKs), and protein phosphatases could transmit sucrose signals [21,22]. All these findings show clearly that the questions of sucrose sensors and the sucrose signaling pathway are still open, and future research could investigate whether other actors such as sucrose synthase (a sucrose-degrading enzyme) [23] or BZR1-BAM (a transcription factor containing a non-catalytic-amylase (BAM)-like domain [24]) might be part of this mechanism.

Like sucrose, trehalose 6P (T6P) is a non-reducing disaccharide, synthesized from UDP-glucose (UDPG) and glucose 6-phosphate (Glc6P) by trehalose 6P synthase (TPS), and then dephosphorylated into trehalose by trehalose 6P phosphatase (TPP, Figure 1) [25]. *Arabidopsis thaliana* has 11 TPS and 10 TPP genes [26], and large TPS and TPP gene families are present in other flowering plants [27,28,29]. The T6P pathway has emerged as an important regulatory mechanism in plants that affects cell metabolism, plant growth, and abiotic stress responses [30,31,32,33]. More interestingly, T6P levels appear to follow sucrose levels, which makes it an essential signal metabolite in plants, linking growth and development to carbon availability [34]. These findings led to the proposal of the Suc-T6P nexus model, assuming that T6P is both a signal and a negative feedback regulator of sucrose levels [31,32,35]. This tight relationship between T6P and sucrose contributes to maintaining sucrose levels within an optimal range depending on cell type, developmental stages, and environmental cues [31,32,35].

### 2.2. Hexose-Dependent Pathways

Plant cells possess intracellular and extracellular sugar sensors. In many plants, the regulator of G-protein signaling 1 (RGS1), a seven-transmembrane-domain protein located on the plasma membrane, plays a critical role as an external sugar sensor (Figure 1) [36]. It could function as a plasma membrane sensor or partner responding to changes in glucose, fructose, and sucrose levels [37]. RGS1 proteins deactivate G-protein and sugars accelerates GTP hydrolysis by Gα subunits, and leads to RGS1 phosphorylation by WINK [with no lysine8 (K)] kinase. As a result, RGS1 is internalized by endocytosis, associated with sustained activation of G-protein-mediated sugar signaling [38]. High concentrations of d-glucose rapidly induce RGS endocytosis through AtWNK8 and AtWNK10, whereas low sustained sugar concentrations slowly activate the AtWNK1pathway [39], allowing the cells to respond adequately to high and low intensities of sugar signals.

The first intracellular glucose sensor demonstrated in plants was AtHXK1, an *Arabidopsis thaliana* mitochondrion-associated (type B) hexokinase [40,41,42,43]. AtHXK1 is involved in the regulation of many processes [6,44], and its function as a glucose sensor was confirmed by the characterization of the *Arabidopsis gin2* (*glucose insensitive 2*) mutant [42]. When the metabolic activity of AtHXK1 was uncoupled from its signaling activity, its glucose-sensing function was found to be independent of its catalytic activity [42]. Moreover, AtHXK1 has a nuclear signaling function [43], where it interacts with two unconventional partners, the vacuolar H^+^-ATPase B1 (VHA-B1) and the 19S regulatory particle of the proteasome subunit (RPT5B) to form a hetero-multimeric complex for the recruitment of hypothetical transcription factors. Such a multimeric complex may bind to the promoters of glucose-inducible genes to modulate their expression. The sensing role of AtHXK1 appears to have been evolutionarily conserved and to be shared by HXK in many other species including potato (StHXK1 and 2) and rice (OsHXK5 and 6) [45,46]. In addition, Hexokinase-Like1 (HKL1, a mitochondrion-associated non-catalytic homolog of *AtHXK1*) [47,48] and AtHXK3 (a plastidial hexokinase) [49] may also be part of hexose signaling. Irrespectively of its well-known sensor function, recent studies have revealed new physiological functions of glucose phosphorylation by AtHXK [50,51].

We currently have no evidence of the role of fructokinase (FRK), which catalyzes the irreversible phosphorylation of fructose, in the fructose-sensing process [44]. However, combining cell-based functional screen and genetic mutations, Cho and Yoo (2011) identified the nuclear-localized fructose 1-6-bisphosphatase (FBP/FIS1, Fructose-Insensitive 1) as a putative fructose sensor uncoupled from its catalytic activity [52]. Meanwhile, Li et al. (2011) characterized the ANAC089 transcription factor as a fructose-sensitivity repressor in *Arabidopsis* [53]. Other researchers isolated two FRK-like proteins (FLN1 and FLN2) that are components of the thylakoid-bound PEP (plastid-encoded polymerase) complex in *Arabidopsis thaliana*, regulate plastidial gene expression, and are essential for plant growth and development [54]. It will be interesting to determine whether there is a connection between these different fructose sensors and how these two hexose pathways could interact.

### 2.3. Energy and Metabolite Sensors

#### 2.3.1. SnRK 1: Sucrose Non-Fermenting Related Protein Kinase 1

AtKIN10/AtKIN11/SnRK1 is a serine/threonine kinase that shares high sequence identity with yeast SnF1 and mammal AMPK (5′ AMP-activated Protein Kinase), and is considered as the energy-signaling hub controlling many regulatory proteins [55,56]. The evolutionary conservation of this protein function is demonstrated by its heterotrimeric structure, with one catalytic α-subunit and two regulatory ß and γ subunits [57], as well as the functional complementation of the yeast *snf1* mutant with the rye orthologue [58]. In plants, SnRK1 plays a crucial role in the reprogramming of metabolism, the adjustment of growth and development, and plant responses to different biotic and abiotic stresses [55]. SnRK1 controls the expression of more than one thousand genes coding for transcription factors and proteins involved in chromatin remodeling, and also acts through post-translational regulation of several key metabolic enzymes and certain transcription factors [59,60,61,62,63]. There exists a positive correlation between the expression profile of genes regulated by AtKIN10 and the profile of genes regulated by sugar starvation [61]. These authors reported that the regulation of SnRK1 activity and signaling required the phosphorylation of a highly conserved threonine residue close to the active site in the catalytic α-subunit. The dephosphorylation of SnRK1α by PP2C phosphatases may reverse the activation loop and provide mechanisms for the integration of environmental cues [64]. The complex interplay of SnRK1 and SnRK2/ABA with the clade of PP2C phosphatases has been demonstrated in the ABA signaling pathway, where both types of protein kinases encompass common downstream targets such as different bZIP transcription factors [63]. In addition, the involvement of micro RNAs in SnRK1-mediated signal transduction appears plausible [65]. It is noteworthy that at least part of the gene responses related to the SnRK1 pathway might be independent of HXK1 signaling.

#### 2.3.2. TOR Kinase: A Target of Rapamycin Kinase

TOR-kinase is evolutionarily conserved in all eukaryotic organisms, and plays a central role in the integration of endogenous (energy and nutrient status) and exogenous (environmental factors) signals to modulate growth and development. In plants, TOR-kinase has been identified only as the TORC1 complex, whose organization is similar in animals and yeast. The complex encompasses the TOR partners RAPTOR (kontroller of growth protein 1 (KOG1)/regulatory-associated protein of mTOR) and LST8 (small lethal with SEC13 protein 8) [66]. The crucial function of TOR-kinase lies in the positive regulation of metabolism and growth through the synthesis of proteins [16]. TOR-kinase activity seems to control nitrogen and carbon assimilation, and has a fundamental role in embryogenesis, meristem activity, leaf and root growth, senescence, and life span through the inhibition of autophagy [67,68,69,70]. As a result of TOR activation, growth is resumed, and reserve compounds, starch, lipids, and proteins are stored [71].

Sugars generally induce the activity of TOR kinase [16,69,72,73]. The glucose-enhanced activity of AtTOR was evidenced by the stimulation of root meristem activity [74]. Inversely, specific inhibitors of AtTOR reduced root growth. TOR activates glycolysis to enhance carbon skeleton production, which is further needed for amino acid and protein synthesis. AtTOR interaction with the RAPTOR protein is involved in the regulation of the ribosomal protein kinase S6K1, which represses the cell cycle by phosphorylating the Retino-Blastoma-Related (RBR) protein under unfavorable conditions [75]. Furthermore, it has been suggested that RBR is involved in the transition from heterotrophic to autotrophic plant development, which requires the expression-inhibition of cell cycle genes, and the persistent inactivation of late embryonic genes through the modification of the epigenetic landscape, namely the trimethylation of lysine 27 of histone H3 (H3K27met3) [76].

Both the SnRK1 and TOR-kinase regulators act antagonistically. Energy and nutrient (C and N) scarcity activates SnRK1 to promote energy-saving and nutrient remobilization processes, while TOR is active under favorable conditions, when nutrients are available to enhance growth, development, and the anabolic metabolism. Such a crosstalk between TOR-kinase and SnRK1 is central for plants to adapt protein synthesis and metabolism to the available resources [77,78,79]. TOR and SnRK1 act downstream of sugar sensing and theirs activities are modulated by the sugar status of plants (Figure 1) [16]. T6P potentially inhibits AtSnRK1, supporting the model that T6P reflects cell sugar availability and promotes growth by repressing SnRK1 activity in sink tissues [31,32,35,80,81,82,83].

#### 2.3.3. The OPPP: The Oxidative Pentose Phosphate Pathway

Specific investigations of sugar sensing, downstream of HXK, highlighted the occurrence of the OPPP in plants (Figure 1). This pathway seems to govern nitrogen and sulfur acquisition in response to the carbon status of the plant [84]. This integration would be critical for the coordination of amino-acid biosynthesis with the availability of these three important components.

### 2.4. Sugar Signaling in the Regulation of Sugar Transporters

*AtSTP1* (*Arabidopsis thaliana* sugar transporter 1) was the earliest-discovered sugar transporter gene regulated by light [85]. Two other STP genes (*AtSTP13* and *AtSTP14*) have been reported to be sensitive to light conditions. They display diurnal regulation, which may result from a direct light effect or from the sensing of photosynthesis-derived sugars [86]. As sugar sensing plays a significant role in the establishment of different developmental stages, sugar transport and signaling appear crucial for these transitions. The latter assumption is corroborated by the changes in the transcriptional control of many sugar transporter genes in distinct mutants such as the Arabidopsis mutant “sweetie”, which is strongly affected in carbohydrate metabolism and displays an upregulated *STP1* gene [87]. The tight connection between sugar signaling and the regulation of sugar transporters fits with the impact of SnRK1 alteration on the expression of several *STPs* (*AtSTP1*, *AtSTP3*, *AtSTP4*, *AtSTP7*, and *AtSTP14*) by transient expression in mesophyll protoplasts [61].

Glucose-dependent down-regulation has been suggested for several STP genes (*STP1*, *STP4*, *STP13*, and *STP14*), but there appears to be different pathways for sugar control of *STP* expression [4,86,88,89,90]. Based on the demonstrated down-regulation of *STP4* and *STP10* in two independent hexokinase1 mutants, it has been suggested that their transcriptional control requires the glucose sensor HXK1. Inversely, *STP1*, one of the genes most repressed by sugars as revealed by large-scale genome analyses, displays glucose-dependent regulation independent of HXK1 [89]. The high responsiveness of *STP1* to sugars is corroborated by the rapid modulation of its expression in response to minor fluctuations of sugar levels (5 mM glucose) [4,88].

## 3. Molecular Mechanisms Involved in Regulation by Sugars

### 3.1. Epigenetic Regulation

Epigenetic mechanisms (DNA methylation, histone posttranslational modifications), as well as small RNAs, histone variants, chromatin remodeling, and higher-order chromatin organization control chromatin structure and thereby influence gene expression. Combinations of these epigenetic marks reflect the active (accessible) and repressive (inaccessible) chromatin state according to the “histone code hypothesis” [91]. A growing body of evidence from yeast and mammals suggests that metabolic signals also play crucial roles in determining chromatin structure [92]. In plants, histones act as metabolic sensors and may affect sensitivity/resistance to glucose and sucrose in link with germination. In *Arabidopsis*, the enzyme encoded by HISTONE ACETYLTRANSFERASE1 (HAC1), was efficiently involved in histone acetylation and protein–protein interactions required for transcriptional activation of gene expression [93,94,95]. Mutations in HAC1 cause a sugar-response defect, and lead to decreased expression of genes, *AtPV42a* and *AtPV42b*, which encode cystathionine-β-synthase (CBS) domain-containing proteins that belong to the PV42 class of γ-type subunits of the plant SnRK1 complexes [96]. This pioneer finding resulted from a screen for sugar-insensitive mutants. It highlights the fact that *hac1* mutants are resistant to high glucose and sucrose concentrations. The authors discussed the possible role of this histone acetyltransferase in the plant sugar response through long-term effects of the nutritional status on the expression of a specific set of genes. The detailed phenotyping of these mutants revealed delayed flowering times possibly related to the indirectly increased expression of the central floral repressor FLC (FLOWERING LOCUS C) by HAC1 [97,98], and a low productivity, namely a reduced number of seeds per silique [96]. The importance of sugar levels for the induction of flowering [99,100,101] suggests a putative involvement of HAC1 in the link between the sugar response and flowering time.

The fine-tuning of the cell fate between proliferation and differentiation in shoot and root apices relies on the balance of antagonistic effects of auxin and cytokinins, but also on glucose and light. All these signals are integrated by TOR kinase. Furthermore, the mutation of transcriptional corepressor TOPLESS, which is required to repress root formation at the shoot pole in late embryogenesis, may be suppressed by a mutation in HAC1. These data suggest that the stable switching-off of auxin-inducible genes (i.e., prevention of their activation), needs to be done through chromatin remodeling [102]. We may also speculate that epigenetic regulation seems required in the complex interplay between metabolic and hormonal signaling. New data arguing in favor of this statement concerns a small family of proteins interacting with ABA INSENSITIVE 5 (ABI5), named (ABI5)-binding proteins AFPs. They may interact with the TOPLESS co-repressor to inhibit ABA- and stress-responses, conferring strong resistance to ABA-mediated inhibition of germination in *Arabidopsis* overexpressing lines [103]. Using a yeast two-hybrid system, the authors demonstrated the direct interaction of AFP2 with histone deacetylase (HDAC) subunits and thereby suggested the possible involvement of some AFPs in the regulation of gene expression through chromatin remodeling.

A new histone modification was reported recently, the *N*-acetylglucosamine (GlcNAc) can be O-linked to the serine 112 of H2B by the enzyme O-GlcNAc transferase (OGT) and produces the O-GlcNAcylation mark [104]. The activated sugar UDP-GlcNAc (Uridine diphosphate *N*-acetylglucosamine) acting as co-substrate, is synthesized from extracellular glucose via the hexosamine biosynthesis pathway. For a first time, the activity of a histone-modifying enzyme is directly linked to the extracellular glucose concentration. Fujiki et al. demonstrated that O-GlcNAcylation of histone H2B at Ser112 fluctuates in response to extracellular glucose. Through a genome-wide analysis, they revealed that H2B Ser112 O-GlcNAcylation was frequently located nearby transcribed genes, suggesting that histone H2B O-GlcNAcylation facilitates gene transcription [104]. GlcNAc is a key signaling metabolite that can coordinate the glucose and glutamine metabolisms in cells through glycosylation of growth factor receptors [105]. Although the transcriptional implication of histone GlcNAcylation remains to be fully elucidated, it is likely that a flux through the hexosamine biosynthesis pathway affects the levels of this novel epigenetic mark. Therefore, nutrient sensing by OGT may be pivotal in the modulation of chromatin remodeling and in the regulation of gene expression [106,107].

In *Drosophila melanogaster*, OGTs act as part of chromatin-remodeling complexes (CRCs) called polycomb group (PcG) proteins [108,109]. Switch/Sucrose-Nonfermenting-(SWI/SNF) type CRCs are constituted of a central Snf2-type ATPase associated with several non-catalytic core subunits evolutionarily conserved in eukaryotes. In *Arabidopsis*, the BRAHMA (BRM) ATPase and SWI3C CRC subunits act within a common complex to fulfill most of their functions, i.e., regulation of transcription, DNA replication and repair, and cell cycling. In addition, the *swi3c* mutation inhibits DELLA-dependent transcriptional activation of GIBBERELLIN-INSENSITIVE DWARF1 (GID1) GA-receptor and GA3ox (active GA biosynthesis) genes [110]. SWI3C also interacts with the O-GlcNAc transferase SPINDLY (SPY) required for the functioning of DELLAs in the GA-response pathway. Physical protein–protein interactions of SWI3C with DELLA and SPY have been demonstrated and further supposed to be required for certain DELLA-mediated effects such as transcriptional activation of the GID1 and GA3ox genes. The established pivotal role of DELLA proteins as a hub in the hormonal crosstalk between gibberellins, auxins, abscisic acid, ethylene, and brassinosteroids has been further supported by their direct physical interaction with SWI/SNF-type CRCs [111]. In the same context, it appears tantalizing to decipher the roles of sugar signaling because it interacts so tightly with hormone signaling. Moreover, it is possibly involved in the linking of the energy metabolism with epigenetic regulation through the expression of specific sets of genes in plant growth, development, and stress responses.

### 3.2. Transcriptional Regulation 

Sugars affect the expression level of many genes in plants. Approximately 10% of *Arabidopsis* genes are sugar-responsive [4,112]. Based on the functional characterization of many sugar-regulated plant promoters, different cis-acting elements have emerged as crucial components of sugar-regulated gene expression. Such cis-acting elements transduce signals of sugar starvation [113,114] or sugar supply [115,116,117,118,119]. The GC-box (GGAGAACCGGG), G-box (CTACGTG), TA-box (TATCAA), and their variants are associated with sugar starvation promoting the expression of α-Amy3 (α-amylase 3, an endo-amylolytic enzyme catalyzing starch degradation in higher plants) [113,114]. By contrast, Sporamin Promoters 8 [SP8a (ACTGTGTA) and SP8b (TACTATT)] and SUgar REsponsive-elements SURE1 (AATAGAAAA) and SURE2 (AATACTAAT) are involved in sugar induction of sporamin and patatin expression, respectively [117,120]. Other cis-acting elements such as the B-box (GCTAAACAAT), the CGACG element, the TGGACGG element, and the W-box (TGACT), S box (CACCTCCA), and TTATC element also participate in plant sugar signaling [117,118,119,121]. Sugar-responsive transcription factors include members of the ERF/AP2 [122], MYB [123], WRKY [124], and bZIP [125] families (Table 1). The first to be identified were SPF8 and SUSIBA2 (SUgar SIgnaling in BArley) [120,124], two members of the plant-specific WRKY family [126]. SUSIBA2 has a high relevance in plant sugar signaling because it is specifically sugar inducible, binds to SURE and W-box elements of the isoamylase (iso1) promoter, a key amylopectin-synthesizing enzyme, and induces carbohydrate accumulation in barley endosperm [124]. This regulation is biologically important, and makes the activity of SUSIBA2 a major component of the high sink strength of seeds. In agreement with this, SUSIBA2-expressing rice preferentially reallocates photosynthates towards aboveground tissues (stem and seeds); this offers sustainable means of increasing starch contents for food production and limits root-related greenhouse gas emission during rice cultivation [127]. In barley leaves, SUSIBA1 and SUSIBA2 are involved in an antagonistic SUSIBA regulatory network that ultimately leads to the coordination of starch and fructan synthesis in response to sugar availability [128]. Low sucrose concentrations generally upregulate SUSIBA1 expression, which acts as a repressor of both fructan synthesis and SUSIBA2 expression, while high sucrose levels induce SUSIBA2 expression, which stimulates starch accumulation in leaves but represses SUSIBA1 expression. This intertwined regulation shows that SUSIBA2 expression depends on the competitive binding of SUSIBA1 (repressor)/SUSIBA2 (inducer) to the target site (W-box) of the SUSIBA2 promoter, building up an autoregulatory system for the sequential regulation of starch and fructan synthesis [128]. Noteworthy, we do not know as yet whether the SUSIBA-related function in sugar signaling is evolutionarily conserved in eudicots. Besides the WRKY family, transcription factors from the bZIP and MYB families are also involved in sugar-regulated gene expression. bZIP is one of the largest transcription factor families in higher plants, characterized by a leucine zipper and a basic region necessary for their specific binding activity to various ACGT elements in plant promoters [129]. This bZIP family regulates the expression of genes involved in an array of plant biological processes and environmental stresses [130,131,132,133,134,135,136]. Early investigations based on transcriptomic analysis identified 10 sugar-responsive bZIP transcription factors in *Arabidopsis*. Amongst them, *AtbZIP1* is transcriptionally and post-translationally repressed by glucose through a hexokinase-dependent pathway [4], which explains its high expression under conditions of sugar and energy depletion [137]. Besides its negative role in early seedling growth, *bZIP1* acts as a master regulator gene for the sugar-signaling pathway: most of the genes identified as *AtbZIP1*-regulated are also sugar sensitive, and it could probably mediate sugar signaling by binding to the C- or G-boxes in the promoters of its target genes [125,137]. Unlike SUSIBA2, AtbZIP1 is involved in other signaling pathways, especially those elicited by light, nutrients, and stress signaling [125,138]. Some transcription factors of the MYB family also mediate sugar signaling, as shown for sugar-induced anthocyanin biosynthesis. Out of the transcription factors involved in this process, MYB71/PAP1, whose expression is upregulated by sucrose availability [139], stimulates the anthocyanin biosynthesis pathway [140,141] through sugar signaling. In fact, the myb71/pap1mutant is defective in sucrose-induced DRF (dihydroflavonol reductase) expression; DRF encodes a key enzymatic function in anthocyanin accumulation, and the alteration of MYB71/PAP1 protein-binding activity related to natural variation in *Arabidopsis* accessions impairs sugar inducibility [13]. Other MYB transcription factors contribute to the sugar-dependent regulation of α-amylase expression. In rice suspension cells and barley aleurone, three structurally related OsMYB transcription factors (OsMYB1, OsMYB2, and OsMYB3) bind to the same target site (a TA-box), share overlapping sequences, but display different biological functions, with a prominent role of OsMYB1 in sugar-starvation-induced α-amylase gene expression [123]. These OsMYBs are also involved in GA-induced expression of α-amylase. This raises the question of their multifunctional roles in many signaling pathways [123]. More recently, Chen et al. (2017) identified two homologs of OsMYB1 in *Arabidopsis* (MYB1 and MYB2) that also recognize the sugar-response element TA-box, but play opposite roles in glucose signaling in *Arabidopsis* [142].

### 3.3. Post-Transcriptional Level

Gene post-transcriptional regulation plays a determining role in plant growth and represents a powerful strategy for plants to flexibly adapt their growth and development to endogenous and exogenous stimuli. This could involve regulation of the rate of mRNA turnover. Extensive investigations have reported that RNA-binding proteins (RBPs) regulate many aspects of RNA processing [143]. In rice cell cultures, nuclear run-on transcription. and mRNA half-life analyses revealed that sugar starvation led to reduced mRNA transcription rates and stability of a large set of genes [144]. Using microarray experiments, Nicolai et al. (2006) identified 268 mRNAs and 224 mRNAs under transcriptional and post-transcriptional regulation, respectively, in response to sugar starvation [145]. Most of them were sugar-starvation-repressed and related to the plant cell cycle and plant cell growth, supporting a rapid adaptability of cell metabolic activity to a low sugar status. Another example that links sugar availability to mRNA destabilization resides in the sugar-induced instability of α-Amy3 [146], probably through the 3′UTR sequence of α-Amy3 RNA [147,148]. Although such post-transcriptional regulation is involved in sugar-controlled mRNA stability, much remains to be understood about the molecular mechanisms involved in this process. UPF RNA helicase, a key component of NMD (nonsense-mediated RNA decay) in *Arabidopsis*, takes part in the sugar-signaling pathway [149], as demonstrated by the low-β-amylase1 (*lba1*) phenotype of the AtUP-1 RNA helicase mutant [150]. Consistently, many sugar-inducible genes were upregulated when the *lba1* mutant was complemented with the wild type AtUPF1, suggesting that AtUPF RNA helicase might optimize sugar inducibility [149]. Similarly, AtTZF1, which encodes an *Arabidopsis thaliana* tandem zinc finger protein, is sugar responsive and might confer sugar-dependent post-transcriptional regulation [151]. This family of proteins plays a critical role in plant growth, development, and stress responses, probably via regulation RNA processing [152].

An emerging process behind sugar-dependent post-transcriptional regulation is mediated by miRNA. miRNAs are regarded as the most important gene regulators that hinder much of gene expression [153]. Many studies have demonstrated that sugars induced the juvenile-to-adult transition by repressing the levels of two members of miR156 (miR156A and miR156C) in *Arabidopsis* seedlings [154,155]. miR156 is known to promote leaf juvenility by impeding the function of SPL (SQUAMOSA PROMOTER BINDING PROTEIN-Like) transcription factor [156]. This sugar effect implies the AtHXK1 signaling pathway and T6P [154,157], and occurs cooperatively with the Mediator Cyclin-Dependent Kinase 8 (CDK8) module [158]. All these findings indicate that sugar-dependent post-transcriptional regulation is far from being a neglected mechanism, but rather involves a diversity of complex processes that requires more investment to decipher the regulatory molecular network.

### 3.4. Translational and Post-Translational Regulation

Regulation of mRNA translation can operate at multiple stages, including through regulatory elements in the 5′ and 3′ untranslated regions (UTRs) of mRNA. Upstream of the open reading frame (uORF), 30% to 40% of eukaryotic mRNAs are located in the 5′UTR [159] and involved in many developmental and growth-related processes [160,161]. Such regulation concerns the *Arabidopsis* basic leucine zipper *AtbZIP11*, which orchestrates metabolic reprogramming in response to cellular sugar status and energy deprivation conditions [162,163,164,165]. Under high sucrose conditions, *AtbZIP11* is induced at the transcriptional level, while it is repressed at the translational level [4,112,166,167]. This translational regulation requires the second uORF of *bZIP11* mRNA, named sucrose-induced repression of translation (SIRT), which is evolutionarily conserved among all groups of S1 members [167,168,169,170,171]. Data from frame-shift mutation and amino-acid substitution indicate that sucrose specifically causes ribosome stalling during translation of the second uORF, impeding the translation of the bZIP main ORF [167]. Using a cell-free translation model, Yamashita et al. (2017) demonstrated that SIRT was critical for specific sucrose-induced ribosome stalling, and probably acted as an intracellular sensor of sucrose [172]. In heterotrophic maize suspension cells, Cheng et al. (1999) ascribed a determining role to the length of the 3′UTR sequence of Incw1, a cell wall invertase, in sugar-mediated translational control [173]. Incw1 encodes two transcripts that only diverge in the length of their 3′UTR (Incw1-Small RNA and Incw 1-Large RNA) and are differentially regulated by sugars. The 3′UTR of the Incw1 genes may act as a regulatory sensor of carbon starvation and as a link between sink metabolism and cellular translation in plants.

Sugar signaling can also be mediated by different mechanisms of post-translational regulation. The 14-3-3 proteins are phosphoserine-binding proteins that regulate a wide array of targets via direct protein–protein interactions [174]. They are involved in sugar-mediated post-translational regulation. In suspension-cultured cell models, sugar supply promotes both protein phosphorylation and their interaction with 14-3-3 proteins, while sugar depletion induces the dissociation of this protein complex, ultimately leading to selective degradation of these 14-3-3-regulated proteins [175]. This coordinated post-translational regulation could establish a new steady-state balance of metabolic activity adapted to sugar starvation conditions. Energy depletion (sugar starvation) due to the application of 2-deoxyglucose, a powerful blocker of the glycolysis pathway [74], led to the inhibition of key metabolic enzymes [60] related to their coordinated phosphorylation by SnRK1 and recognition by 14-3-3 proteins [57,59,60]. More recently, Okumura et al. (2016) demonstrated that sugar accumulation in photosynthetic leaves induced the activation of the plasma membrane H^+^-ATPase through the phosphorylation of the penultimate threonine of the C-terminal region and the binding of 14-3-3 proteins [176,177]. Accordingly, light-induced phosphorylation of H^+^-ATPase was strongly suppressed in mutants impaired in endogenous sugar accumulation. Such activation of H^+^-ATPase may stimulate sucrose export from leaves and thereby avoid inhibition of photosynthesis by high sugar accumulation [178,179]. Additional mechanisms of sugar-elicited posttranslational regulation come from the regulation of AGPase (ADP gluco-pyrophosphorylase), a key enzyme of the starch biosynthesis pathway [180]. Starch synthesis can increase via allosteric activation of AGPase, as a result of a higher 3PGA (3-phosphoglycerate) to Pi (inorganic phosphate) ratio [181] and/or through its post-translational redox activation in response to light or high sugar treatment in *Arabidopsis* [182,183,184,185]. This latter regulation is dependent on T6P [186,187]. All these findings show that post-transcriptional regulation is prevalent for sugar signaling, and extensive investigations are required to get more information about the molecular mechanisms and the relevance of additional post-transcriptional mechanisms such as sumoylation, protein–protein interactions, or unfolded cytoplasmic proteins.

## 4. Crosstalk between Sugar and Hormone Signaling

### 4.1. Sugars and Auxin

**Figure 2:**
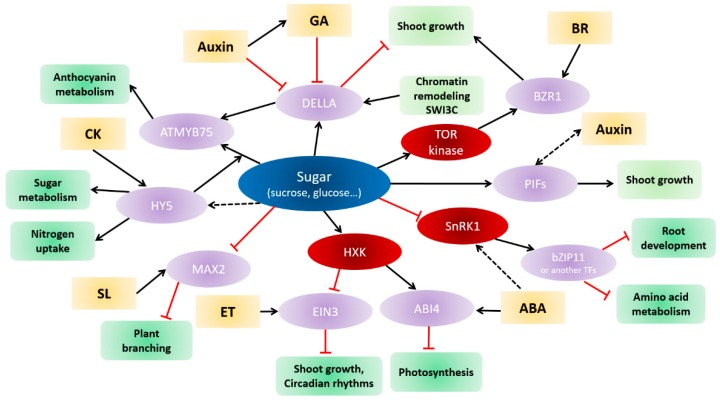
Schematic representation of the crosstalk between the sugar and hormone signaling pathways (orange frame) in the regulation of certain physiological processes (green frame). Light-purple circles represent hub regulators, including transcription factors (ABI4: ABA insensitive 4; ATMYB75/PAP1; bZIP11; EIN3: ETHYLENE-INSENSITIVE 3; HY5: Elongated Hypocotyl 5: PIFs: Phytochrome interacting factors), F-box (MAX2: MORE AXILLARY 2) and key regulators of the hormone signaling pathways (DELLA and BZR1: BRASSINAZOLE RESISTANT). Red circles represent glucose sensors (HXK: hexokinase) and energy sensors (SnRK1: Sucrose non-fermenting related kinase 1; TOR-kinase: Target of Rapamycin kinase). ABA: Abscisic acid; BR: Brassinosteroid; CK: Cytokinin; GA: Gibberellin; SL: Strigolactone. Black arrows indicate stimulating effects and red blunts indicate repressing effects.

Auxin has been chemically identified as indole-3-acetic acid (IAA). It plays a pivotal role in almost all processes of plant growth and development [188,189,190]. Growing evidence suggests a crosstalk between sugar and auxin that involves metabolism [191,192,193], transport [194,195,196,197], and signaling pathways [198,199,200,201]. The first link between sugar and auxin came from *Arabidopsis* hypocotyl explants of *hxk*/*gin2*, which is insensitive to auxin induction of cell proliferation and root formation [42]. Accordingly, auxin-resistant mutants (*axr1*, *axr2*, *tir1*) are insensitive to high glucose concentrations [42]. Sugar and auxin represent a highly complex and central signaling network in various aspects of plant development, including cell proliferation, cell expansion, cell differentiation, hypocotyl elongation, or anther development [179,202,203,204,205,206]. A novel allele of the hookless1 gene (*hls1*) was found resistant to both sugar and auxin responses in excised leaf petioles, suggesting that it may be part of the negative effects of auxin on sugar-responsive gene expression [199]. One of the best examples is their major contributing role in root system architecture, whereby sugar works individually or cooperatively with auxin [200,203,207,208,209]. Transcriptomic experiments on seedling roots revealed that glucose regulates the expression of many auxin-related genes (e.g., YUCCA, TIR1); more interestingly, a number of these genes are insensitive to glucose alone, but become glucose-responsive in the presence of auxin (e.g., AUX/IAA) [200]. In addition, exogenous glucose supply enhances defects in the induction of lateral root growth, root hair elongation, and gravitropism in mutants of auxin sensing (*tir1*) and signaling (*axr2* and *axr3*), suggesting that glucose-regulated root architecture may occur through auxin-based signal transduction [200]. Gonzali et al. (2005) showed that auxin and turanose, a non-metabolizable analogue of sucrose, regulated the expression of the WOX5 transcription factor, a Wushel-related homeobox gene required for sustaining local auxin maxima in the root apical meristem [198]. Additional insights into the interactions between auxin and sugar come from the elegant work conducted in *Arabidopsis* by Weiste et al. (2017): they demonstrated a new function of S_1_bZIP11-related TFs (bZIP1,-11 and -44) as negative regulators of auxin-mediated primary root growth (Figure 2) [165]. These transcription factors are downregulated by sugar and upregulated by low energy levels through SnRK1 kinase activity [61,162,167,170,171,210,211]. In this context, bZIP11 and closely related TFs directly up-regulate the expression of IAA3/SHY2, a key negative regulator of root growth. By repressing transcription of PIN1 and PIN3—major auxin efflux facilitators—IAA3/SHY2 restricts polar auxin transport (PAT) to the root apical meristem and thereby blocks auxin-driven primary root growth. These findings support that *bZIP11* and closely related transcription factors are a gateway for integrating low-energy-related stimuli into auxin-mediated root growth responses [165]. A close interaction between bZIP11 and auxin signaling was also demonstrated when the highly homologous bZIP11-related TFs were found to quantitatively modulate auxin-responsive gene expression by recruiting the histone acetylation machinery to target promoters [212]. Repression of PIN1 accumulation and consequently reduction of PAT in roots explains the inhibition of root elongation in response to high glucose concentrations, through *ABI5* (ABA insensitive 5) [213]. Part of the glucose and auxin interplay in root growth and development is thought to result from the glucose-dependent activation of the heterotrimeric G-protein [214] and the auxin-activated TOR-kinase signaling pathway [215]. Based on genetic analysis, Raya-González et al. (2017) proposed a new mechanism involving MED12 or MED13, two subunits of the MEDIATOR complex responsible for the connection of RNA polymerase II to specific transcription factors [216], and thus controlling gene transcription [217]. The loss-of-function mutants *med12* and *med13* displayed short and thin primary roots associated with decreased auxin responsiveness and a reduction of cell proliferation and elongation in primary roots. This behavior was fully alleviated by exogenous sugar. Further analysis supports that MED12 and MED13 can operate as positive linkers of sugar sensing to the auxin response pathway, and that MED12 acts upstream of AUXIN RESISTANT 1 (AUX1), an auxin influx carrier central for the spatio-temporal transport of auxin within the root tissue. An interesting connection between sugar signaling and polar auxin transport also results from hypocotyl elongation; it involves the PIF (Phytochrome interacting family) transcriptional regulators (Figure 2). PIFs (e.g., PIF4) induces the expression of many auxin biosynthesis genes (e.g., YUCCA) by binding to their promoter [218], and its upregulation by sucrose leads to auxin accumulation and thereby to hypocotyl elongation [195]. The regulation mechanism between sugar and PIF seems to be complex: other works showed that PIFs could act as negative regulators of sugar-induced IAA biosynthesis [196], and that the auxin response acts upstream of PIFs [197]. Additionally, the role of PIFs in linking sugar signaling to auxin accumulation is central during another development in response to high temperature [204]. Although the sugar and auxin interplay plays a coordinated role in the control of many plant developmental processes, extensive investigations are still required to understand whether these so-far described pathways are interconnected or correlated with each other.

### 4.2. Sugar and Cytokinins 

Cytokinins (CKs) are underlying factors of the regulation of plant development. They influence many central processes including cell proliferation, source/sink relationships, leaf senescence, apical dominance, root growth, nutritional signaling, and responses to abiotic and biotic stresses [219,220,221,222,223]. Many examples indicate that CKs and sugars can cross-influence their metabolism and transport [193,222,224,225,226,227,228,229], which may impinge on signaling pathways. Based on transcript profiling of *Arabidopsis* seedlings after glucose and cytokinin treatment, Kushwah and Laxmi (2014) showed that glucose and CKs acted both agonistically and antagonistically on gene expression, and glucose had a strong effect on genes involved in cytokinin metabolism and signaling [229]. The first direct interplay between sugar and CKs came from the characterization of the hxk1/gin2 mutant, which displays decreased sensitivity to sugar and increased sensitivity to CKs [42], suggesting an antagonistic effect of CKs. In accordance with this, a constitutive CK response mutant was insensitive to high glucose-dependent seedling development repression [230], and a CK receptor mutant (ahk3) exhibited CK resistance but increased sucrose sensitivity during plant growth assays [231]. The cytokinin-resistant (cnr1) mutant exhibited a number of altered auxin responses as well as hypersensitivity to sugars, as evidenced by high levels of chlorophyll and anthocyanins as compared to the wild type [232]. Further proof came from the fact that sucrose downregulates WPK4, that is activated by CK and encodes a putative protein kinase belonging to the SnF1 kinase subfamily [233,234]. Other responses rather revealed synergistic effects. In *Arabidopsis* seedlings, CKs and glucose positively regulate root elongation via an HXK1-dependent pathway [235], involving *Arabidopsis* cytokinin receptor AHK4 (ARABIDOPSIS HISTIDINE KINASE 4) and three *Arabidopsis* type-B response regulators (ARR1, ARR10, and ARR11) [235,236]. In that case, glucose only boosts CK-dependent root elongation, which occurs upstream of the effect of auxin on root development [235,236]. CKs and sugar also play important roles in the regulation of different cell cycle phases, including the G1/S transition via the sucrose induction of *cycD3* expression [15] and the G2/M transition [237]. Another synergistic action between CKs and glucose resides in their combinatorial effect on anthocyanin accumulation in *Arabidopsis* leaves: sugar-induced anthocyanin biosynthesis is enhanced by CKs via the redundant action of three type-B ARRs (ARR1, ARR10, and ARR12) [238]. This signaling cascade involves transcriptional upregulation of MYB75/PAP1 by LONG HYPOCOTYL 5 (HY5) (Figure 2) [239].

In *Arabidopsis*, a balance between the antagonistic effects of auxin—which mediates cell division—and CKs—which mediate cell differentiation—establishes the root meristem [240]. Inversely, in the shoot meristem, CKs seem to promote the proliferation of stem cells and inhibit their differentiation, whereas auxin triggers organ primordia initiation [102]. It is noteworthy that the activation of root and shoot apical meristems relies on distinct glucose and light signals [241]. The authors provide arguments that glucose is required and sufficient to activate TOR kinase in the root apex, whereas neither glucose nor light alone can efficiently activate this central integrator of the cell nutrient and energy status in the shoot apex.

### 4.3. Sugars and Strigolactones 

Strigolactones (SLs) were first considered as rhizosphere-signaling molecules that promote the germination of root-parasitic weeds and the symbiotic interactions between plants and soil microorganisms [242,243,244]. Since then, SLs have been revealed as mobile phytohormones controlling plant development and plant adaptation to environmental stress [245,246,247,248,249,250,251,252,253]. In addition, genes involved in SL biosynthesis and signaling are known in many plants [254]. Only a few studies have addressed how sugar signaling and SL signaling interplay to regulate plant functioning. Wu et al. (2017) showed that an *smxl4*/*smxl5* double mutant of *Arabidopsis* caused defective phloem transport of sugar and enhanced starch accumulation [255]. Li et al. (2016) reported that both mutants of *MAX1* and *MAX2*, involved in SL biosynthesis and signaling, respectively, were hyposensitive to glucose repression of seedling establishment; this suggests that this repression is under the cooperative effect of SLs and glucose, probably via the hexokinase-independent pathway [256]. Conversely, an antagonistic effect between the sugar- and SL-signaling pathways has been reported for plant branching. In that case, SLs act as inhibitors while sugars act as inducers [257]. In accordance with this, *Arabidopsis* plants overexpressing cyanobacterial fructose-1,6-bisphosphatase-II in the cytosol exhibited an over-branching phenotype associated with high sugar contents and repression of *MAX1* and *MAX4*, two SL-biosynthesis genes [258]. These findings fit with those reported by Barbier et al. (2015) in Rosa sp. buds, in which both metabolizable and non-metabolizable analogs of sucrose or hexoses down-regulated *MAX2* expression, and this effect coincided with the ability of buds to grow out [193]. Further investigations should be led to ascertain the components through which these two signals interact.

### 4.4. Sugars and Gibberellins 

Gibberellins (GAs) constitute a large group of molecules with a tetracyclic diterpenoid structure. They function as plant hormones and influence photosynthesis, the carbohydrate metabolism, plant growth (cell and stem length elongation) and development processes (germination, flowering etc.), and responses to biotic and abiotic stresses [259,260,261,262,263,264,265,266,267,268]. GAs directly regulate the plant carbon status through their effect on photosynthetic activity [269,270,271] and sugar metabolic activity [272,273,274,275,276,277,278,279]. Sugar can also affect the GA metabolism in different biological contexts [49,280]. A first direct interaction between sugar and GA signaling was revealed by the sugar-insensitive1 (*sis1*) mutant, which was also insensitive to gibberellins during seed germination [281], suggesting a cooperative effect between sugars and GAs. However, other data provide evidence for an antagonistic crosstalk. Sugar and GA signaling compete to regulate the expression of α-amylase in many cereal seeds [282,283,284,285], which involves spatiotemporal transcriptional regulation of GAMYB [285,286]. Li et al. (2014) reported that sucrose stabilizes DELLA proteins [287], supporting the importance of GAs in dark-induced hypocotyl elongation [288] and their negative effect on the sucrose-dependent induction of the anthocyanin biosynthesis pathway [289]. DELLA proteins are central repressors of the GA response [290]. In this context, Loreti et al. (2008) showed that GA repressed the expression of several sucrose-induced genes involved in anthocyanin synthesis, and this repressive effect was noticeably absent in *gai*, a mutant expressing a stabilized DELLA protein [289]. DELLA acts by inducing the upregulation of PAP1/MYB75 [291], a sucrose-induced transcription factor that mediates anthocyanin synthesis (Figure 2) [13]. DELLA proteins could be one of the main convergent actors of the hormonal and sucrose-dependent molecular networks.

### 4.5. Sugars and Ethylene

Ethylene regulates a host of plant processes, ranging from seed germination to organ senescence and plant responses to biotic and abiotic cues [292,293,294]. In higher plants, the pathways of ethylene metabolism and signaling are well established [295]. As evidenced by genetic and phenotypic analyses of many *Arabidopsis* mutants, there is a tight, but generally antagonistic, interaction between sugar and ethylene signaling. Mutants displaying nonfunctional ethylene receptors (*etr1*, *ein4*) or alteration of signal transduction proteins (*ein2* and *ein3*), are hypersensitive to sugar-mediated photosynthesis repression, while constitutive triple response 1 (*ctr1*), a negative regulator of ethylene signaling, is glucose insensitive [281,296,297,298]. Consistently, the ethylene-insensitive *etr1* and *ein2* mutants both exhibit *glo* (glucose-oversensitive) phenotypes, whereas the constitutive ethylene signaling mutant *ctr1* is allelic to *gin4*, a glucose-insensitive mutant [296,299]. Such an antagonistic interaction explains the ability of glucose to stimulate the degradation of ETHYLENE-INSENSITIVE3 (EIN3), a key positive transcriptional regulator in ethylene signaling, through the hexokinase-signaling pathway, and thereby promote plant growth [298]. The antagonistic interplay between the sucrose and ethylene pathways is involved in sustaining sugar-dependent circadian rhythms in darkness through post-transcriptional regulation of the circadian oscillator GIGANTEA (GI) [300]. According to these authors, sugars maintain circadian rhythms in the evening/night transition by stabilizing the GIGANTEA (GI) protein depending on the activity of the F-Box protein ZEITLUPE (ZTL), and by destabilizing EIN3 (Figure 2) [300]. The ethylene- and sugar-signaling pathways can also work cooperatively: both ethylene-insensitive (*ein2-1*) and ethylene-constitutive response (*sis1/ctr1)* mutants displayed only a slight modification of sugar-dependent chlorophyll accumulation and growth, but higher impairment of the sugar-induced tolerance to atrazine (an inhibitor of electron transport in photosystem-II photosynthesis) [301]. It appears that the mechanism of sucrose-dependent protection against atrazine requires active ethylene signaling and may rather involve a hexokinase-independent pathway.

### 4.6. Sugars and Abscisic Acid 

Abscisic Acid (ABA) regulates many plant adaptive responses to environmental constraints [302,303,304]. Among plant hormones, ABA is one of major importance in the interaction with sugar signals since many mutations affecting sugar sensing and signaling are allelic to genes encoding components of the ABA synthesis or ABA transduction pathways. In *Arabidopsis*, ABA-deficient (*aba2*, *aba3*) and ABA-insensitive (*abi4*) mutants are allelic to glucose insensitive1 (*gin1*)/impaired sucrose induction 4 (*isi4*)/sugar insensitive1 (*sis1*) [305,306], and *gin6*/*isi3*/*sis5*/*sun6* [122], respectively. The *abi8* mutants are resistant to glucose-mediated developmental arrest of wild-type seedlings [307]. The crosstalk between sugar and ABA can involve synergistic effects with ApL3 (ADP pyrophosphorylase large subunit) [306,308], ASR (ABA, stress and ripening-induced protein) in grape [309] and many photosynthesis-related genes [121,310,311]; but it can also involve agonistic effects, e.g., on the expression of Rab16A [312], Amy3D [115], and OsTIP3.1, a tonoplast intrinsic protein [313]. ABI4, a transcription factor of the AP2/ERF family, plays a prominent role in glucose and ABA signaling [121,122,314,315]. Many investigations underline the key role of SnRK1 in glucose and ABA signaling, since plants over-expressing SnRK1.1 displayed hypersensitivity to glucose and ABA during early seedling development [316], and SnRK1 was required for ABA-mediated maturation of pea seeds [317]. In addition, mutants disrupted in the three SnRK1 subunits displayed full ABA insensitivity, supporting that SnRK-mediated protein phosphorylation is necessary for all aspects of ABA functioning [318]. Exogenous ABA application resulted in the fine-tuning of SnRK1 activity [319]. Overlapping between the ABA and sucrose pathways was also confirmed by the phenotype of *aip* (a null mutant of AIP1), a member of group A PP2C serving as a positive actor in ABA signaling. This mutant exhibited lower sensitivity to ABA and glucose during seed germination and early seedling development [320]. More recently, Carvalho et al. (2016) identified a negative regulator of the glucose signaling pathway corresponding to the plant-specific SR45, belonging to the highly conserved family of serine/arginine-rich (SR) proteins [321]. Phenotypic and molecular characterization of the *sr45-1* mutant revealed hypersensitivity to glucose during early seedling growth, and over-induction of ABA-biosynthesis and ABA-signaling genes in response to glucose as compared to the wild type.

### 4.7. Sugars and Brassinosteroids 

Brassinosteroids (BRs) are a class of polyhydroxylated sterol derivatives that regulate many plant growth processes [322,323,324,325,326]. Glucose affects BR biosynthesis, perception, transduction, and homeostasis [327,328,329]. On the other hand, BRs influence CO_2_ assimilation, metabolism, and sugar fluxes [330,331,332,333,334,335,336,337,338,339]. BRs and sugars interplay cooperatively to regulate certain developmental processes, including etiolated hypocotyl elongation and LR development in *Arabidopsis* seedlings [340,341,342]. Analysis of glucose and BR sensitivity in both hexokinase-dependent and hexokinase-independent pathways complete this picture, providing evidence that glucose and BRs act via HXK1 pathway and BRs act downstream of this glucose sensor [329,341,342]. BZR1 (BRASSINAZOLE RESISTANT 1) protein can represent a converging hub between sugar and BR signaling (Figure 2) [341,343], and its stability is positively and synergistically controlled by TOR-dependent and BR signaling pathways in hypocotyls [343]. According to these authors, such TOR-mediated regulation allows carbon availability to control the hormonal growth promotion programs, ensuring a supply-demand balance in plant growth. Synergetic overlapping between sugar and BR signaling might also take place in the regulation of floral signal transduction, with a downstream effect of BZR1 and BZR2 [263]. This assumption is linked to the fact that many BR-deficient mutants (*brs1*, *det2*, *cpd*, *bls1*) display a late-flowering phenotype, and the *bls1* (*brassinosteroid*, *light and sugar1*) mutant is hypersensitive to sugars [344].

### 4.8. Crosstalk between Sugar and Nitrate Signaling

#### 4.8.1. Interactions between Sugars and Nitrogen

The control of C and N interactions involves various endogenous signals, NO_3_^−^ and NH_4_^+^ ions, amino acids, as well as sugars issued from the carbon metabolism [345,346,347,348,349]. Nitrate acts as a positive signal for the induction of proper sugar uptake and assimilation, while the metabolites resulting from sugar assimilation—e.g., glutamate, glutamine, aspartate, and to a lesser extend glycine and serine—serve as negative signals [350,351,352,353,354,355,356]. NR (nitrate reductase) and PEPc (phosphoenolpyruvate carboxylase) are two enzymes that link primary N and C assimilation in plants [357]. The interdependence of the C and N metabolisms supports their roles in the regulation of gene expression that can occur at the cell, organ, or whole plant levels [358]. Furthermore, nitrate uptake is enhanced by the CO_2_ level through the availability of carbohydrates, and inversely a reduction of carbon storage by defoliation impairs nitrate uptake [345,349,359,360,361,362,363]. In line with this, the dark-dependent decrease of N uptake is reversed by addition of sucrose, which transcriptionally affects high- and low-affinity nitrate transporters [364]. Moreover, the regulation of nitrate uptake by light and sucrose is strongly linked [365], and NR gene expression appears to be regulated by photosynthesis through glucose synthesis [366]. Taken together, these results strongly suggest that the C/N ratio affects NR and NiR gene expression as well as the related enzyme activities. Nitrogen starvation of wheat seedlings significantly decreased both the transcript levels and enzyme activities of NR, NiR, GS, and GOGAT, while potassium nitrate and ammonium nitrate restored gene expression and the catalytic activities of these enzymes [367]. In *Brassica juncea*, the expression of most of the N-pathway genes is significantly modulated under exogenous supply of sucrose or of sucrose and nitrogen [368]. These data emphasize the importance of C/N ratio signaling in the regulation of gene expression. 

#### 4.8.2. C/N Regulation

Many transcription factors—bZIP, Dof, Nin-like protein 7—as well as proteins such as kinases/phosphorylases are involved in regulating both the carbon and nitrogen metabolisms. Besides TOR kinases, other regulatory hubs between sugars and the nitrogen metabolism have been identified in plants. Among them, Elongated Hypocotyl 5 (HY5) is a bZIP transcription factor involved in a great number of signaling pathways such as hormonal, metabolic, or abiotic stress pathways [369]. It may operate in combination with the circadian rhythm to adjust levels of photosynthetic gene expression in the daytime [370]. HY5 is a shoot-to-root phloem-mobile signal and it mediates the regulation of root growth and nitrate uptake by light [369,371,372]. In the shoot, HY5 promotes carbon assimilation and translocation, whereas in the root it mediates the activation *NRT2.1* expression, supporting the fact that it coordinates plant nutrition and growth in response to fluctuating light environments (Figure 2). Furthermore, HY5 regulates the sucrose metabolism and sucrose movement into phloem cells for shoot-to-root translocation by increasing the expression levels of *SWEET11* and *SWEET12*, two genes encoding sucrose efflux transporters required for sucrose phloem loading [371,373]. Taken together, all these results strongly suggest that HY5 mediates the homeostatic regulation of the whole-plant C status versus the whole-plant N status [371]. Furthermore, the master clock control gene CCA1 targets the transcription factor bZIP1, which itself targets ASN1 involved in asparagine synthesis, and the nitrate transporter 2.1 (NRT2.1) [4,138]. SnRK1 phosphorylates NR [374,375] supporting the impact of both SnRK1 and bZIP1 on nitrogen signaling. Dröge-Laser and Weiste (2018) recently highlighted the importance of bZIPs in the control of metabolic gene expression according to the plant nutritional status [376].

The Dof1 (DNA binding with one finger) transcription factor from maize (*ZmDof1*) up-regulates the expression of PEPc and thereby modulates the C/N network. Overexpression of *ZmDof1* in *Arabidopsis* and potato was accompanied by the promotion of nitrogen assimilation and plant growth under low nitrogen conditions [377,378,379], and up-regulation of multiple genes involved in carbon skeleton synthesis [378]. In agreement with this, expression of *ZmDof1* regulates the expression of genes involved in organic acid metabolism, leading to the accumulation of amino acids and to increased growth under N-limiting conditions. These effects suggest that nitrogen use efficiency (NUE) could also be improved by manipulating the carbon metabolism pathways [363]. An analysis of the *cis*-regulatory element in the promoter of *AtSUC2*, which encodes a companion-cell-specific proton-sucrose symporter, identified a putative binding site for HD-Zip and a binding site for DOF transcription factors [380]. Furthermore, Skirycz et al. (2006) reported the phloem-specific localization of a member of the DOF transcription factor family [381]. In *Oryza sativa*, OsDOF11 modulates sugar transport by regulating the expression of SUT (SUT1, 3, 4, and 5) and SWEET (SWEET11 and SWEET14) genes [382].

Proteins from the Nodule inception-like protein (NIN-Like Protein—NLP) family appear as master regulators of nitrate signaling. NLP7 and NLP6 bind and activate the promoters of several nitrate-induced genes [78,383,384,385,386]. The only known actor of the signaling mechanisms involved in the induction of *NRT2.1* and *NRT1.1* expression by carbon is the OPPP [84,387,388]. Otherwise, NLP7 is involved in the regulation of the gene coding for 6-phosphogluconate dehydrogenase, a key enzyme of the OPPP [385]. Glucose increases NRT2.1 protein levels and transport activity independently of its hexokinase1-mediated stimulation of NRT2.1 expression, demonstrating another possible post-transcriptional mechanism influencing nitrate uptake [387]. These authors also established that photosynthate availability in the form of glucose is coupled to nitrate uptake and assimilation through glucose metabolism by HXK1 in the OPPP, transcriptional control of NRT2.1, and post-translational regulation of NRT2.1 protein levels and transport activities [387].

## 5. Conclusions

The complex processes of plant growth and development rely on the integration of inputs about nutrient availability, the energy status, and the hormonal balance under variable conditions at the level of whole organisms. Sugar signaling downstream of signal-specific sensors (e.g., hexokinase, RGS1, fructose 1,6 bisphosphatase, O-GlcNAc transferase) converge to hub regulators of the nutrient and energy status (SnRK1 and TOR kinase). These key integrators might mediate the balance between the anabolic and catabolic metabolisms, as well as accumulation of reserves versus remobilization of reserves through epigenetic reprogramming, transcriptional/post-transcriptional regulation, ribosome biogenesis, translational activity, and protein modifications. Extensive investigations are required to identify additional hub regulators with original/unexpected functions. In this regard, the relevance of “non-canonical” sensors including histone modifiers, microRNAs, transcription factors, and many others (small peptides etc.) should not be overlooked. The complexity of sugar-responsiveness processes further requires the approaches of systems biology. The diversity of sugar sensors and the emerging transduction pathways are tightly interconnected with the hormone and nitrate signaling networks. At present, there is still a knowledge gap about the interconnections between all these signal transduction pathways, while the identity of the molecular actors involved in convergence points remains mostly unknown.

## Figures and Tables

**Table 1:** The transcription factors that are involved in sugar signaling in *Arabidopsis thaliana.*

Gene ID	Gene Symbol	Transcription Factor Family	Main Process/Function	Binding Site	References
AT1G69780	ATHB13	HD-ZIP	Response to sugar signaling pathways. Control cotyledon and leaf morphogenesis	5′-CAATNATTG-3′	[100,389,390,391]
AT5G28770	AtbZIP63	bZIP	Response to glucose and ABA signaling pathways	5′-CACGTG-3′ ^*^	[392,393]
AT1G45249	ABF2	bZIP	Involved in ABA and glucose signaling pathways. Response to salt stress	5′-CACGTG-3′; 5′-ACGTGKC-3′	[394,395,396,397,398,399]
AT2G40220	ABI4	AP2/ERF	Response to ABA, ethylene, cytokinin, and sugar signaling pathways. Involved in osmotic stress, defense response and root development, and stomatal movement	5′-CACTTCCA-3′	[121,311,400,401,402,403,404,405]
AT5G24800	AtbZIP9	bZIP	Response to sugar signaling pathway	5′-CACGTG-3′	[406,407]
AT4G34590	AtbZIP11	bZIP	Involved in sugar, auxin signaling pathways. Affect root growth and amino acid metabolism	5′-CACGTG-3′	[162,165,167,212,408]
AT3G20770	AtEIN3	EIL	Response to ethylene and sugar signaling pathways	5′-GGATTCAAGGGGCA TGTATCTTGAATCC-3′	[298,409,410,411]
AT2G28350	ARF10	ARF	Involved in auxin, and ABA signaling pathways. Control cell division, seed germination and developmental growth, and root cap development	5′-TGTCTC-3′ *	[412,413,414,415,416,417,418]
AT1G56650	AtMYB75	MYB	Response to sugar, jasmonic acid, auxin, ethylene signaling pathways. Regulation of anthocyanin biosynthetic process and removal of superoxide radicals. Involved in cell wall formation	5′-CACGTG-3′, 5′-ACACGT-3′	[13,239,289,419,420,421,422,423,424,425]
AT2G36270	ABI5	bZIP	Involved in ABA, sugar signaling pathways during seed germination	5′-CACGTG-3′	[103,122,289,426,427,428,429,430,431]
AT2G30470	HSI2	B3	Repressor of the sugar-inducible genes involved in the seed maturation. Plays an essential role in regulating the transition from seed maturation to seedling growth. Involved in embryonic pathways and ABA signaling	5′-CATGCA-3′	[432,433,434,435,436,437]
AT5G49450	AtbZIP1	bZIP	Involved in sugar (nutrients) signaling pathway. Response to salt and osmotic stress	5′-CACGTG-3′	[125,136,138,210,406,438]
AT3G23210	bHLH34	bHLH	Response to glucose and ABA signaling	5′-(GA)n-3′; 5′-CANNTG-3′	[107,406]
AT3G44290	NAC060	NAC	Response to sugar-and ABA signaling cascade	Unknown	[315,439]
AT4G00238	AtSTKL1	GeBP	Involved in mediating certain glucose responses	5′-GCCT-3′	[440]
AT5G08790	ATAF2	NAC	Response to sugar, jasmonic signaling pathways. Seedling photomorphogenesis and leaf senescence	5′-RTKVCGTR-3′ ^*^	[441,442,443,444,445,446]
AT5G56860	GATA21	GATA	Response to Gibberellic acid and sugar signaling pathways. Involved in regulation of nitrogen compound metabolic process, flower development, cell differentiation, and chlorophyll biosynthetic process	5′-GATA-3′	[447,448,449,450,451,452]
AT3G54320	AtWRI1	AP2/ERF	Response to sugar signaling pathway. Involved in triglyceride biosynthetic process, lipid metabolic process, regulation of glycolytic process, and seed development	5′-CACRNNTHCCRADG-3′ ^*^	[453,454,455,456,457,458,459]
AT4G14410	bHLH104	bHLH	Response to sugar signaling pathway and iron homeostasis	5′-(GA)n-3′	[460,461,462]
AT5G64750	ABR1	AP2/ERF	Involved in ABA and sugar signaling pathways. Response to osmotic stress and salt	5′-GCCGCC-3′	[463,464,465]
AT4G00250	ATSTKL2	GeBP	Response to sugar signaling	5′-GCCT-3′	[440]

The code of bind site follows the IUPAC role. * The binding sites are predicted by PlantPAN database (http://plantpan2.itps.ncku.edu.tw/index.html) [466].
